# Engineering soybean with high levels of herbicide resistance with a Cas12‐SF01‐based cytosine base editor

**DOI:** 10.1111/pbi.14356

**Published:** 2024-04-21

**Authors:** Qingfeng Niu, Hongtao Xie, Xuesong Cao, Minglei Song, Xin Wang, Shasha Li, Kang Pang, Yangyang Zhang, Jian‐Kang Zhu, Jianhua Zhu

**Affiliations:** ^1^ School of Life Sciences, Anhui Agricultural University Hefei Anhui China; ^2^ Research Center for Biological Breeding Technology Research Institute of Frontier Science Anhui Agricultural University Hefei Anhui China; ^3^ Bellagen Biotechnology Co., Ltd Jinan Shandong China; ^4^ Institute of Advanced Biotechnology and School of Medicine Southern University of Science and Technology Shenzhen China; ^5^ Shandong Normal University Jinan Shandong China

**Keywords:** soybean, herbicide resistance, cytosine base editor

Soybean (Glycine max (L.) Merr.) is among the most important crops in the world for oil and proteins (Zhang *et al*., 2022). Given that weeds are a major challenge to soybean cultivation, non‐transgenic herbicide‐tolerant soybean varieties are in great demand, particularly in places where growing transgenic soybean is prohibited. The clustered regularly interspaced short palindromic repeats (CRISPR)‐Cas gene editing systems, including base editors built from Cas9 and Cas12 systems, are revolutionary tools in plant breeding, including the breeding for herbicide‐tolerant crops (Sun *et al*., 2016; Wang *et al*., 2019; Zhang *et al*., 2023). Although Cas9 base editor systems have been successfully applied in soybean (Huang *et al*., 2023; Niu *et al*., 2023), the application of Cas12 base editors in soybean breeding has not been reported.

Acetolactate synthase (ALS), a crucial enzyme in the biosynthesis of branched‐chain amino acids, is the target of several important herbicides (Powles and Yu, 2010). Variants of ALS that harbours certain point mutations can confer tolerance to a major group of commercial herbicides. A previous study showed that a variant bearing the P197S substitution in AtALS exhibited herbicide resistance in *Arabidopsis* (Chen *et al*., 2017). The soybean genome contains four *GmALS* paralogues (*GmALS1*‐*4*). Notably, the widely planted non‐transgenic herbicide‐resistant soybean harbours a single‐point mutation P178S in GmALS1, equivalent to P197S in AtALS (Walter *et al*., 2014). Unfortunately, amino acid substitution mutation in individual *ALS* genes cannot avoid herbicide injuries (Walter *et al*., 2014 and this study). Therefore, we explored the use of amino acid substitution mutations in multiple *ALS* genes to produce soybeans with sufficient herbicide resistance for effective weed control. Among the four *GmALS* genes, *GmALS1* and *GmALS3* share 80.6% and 79.0% amino acid sequence identities with *AtALS* respectively. We selected *GmALS1* and *GmALS3* as the targets for generation of herbicide‐resistant soybean by converting C to T at codons P178 of *GmALS1* and P172 of *GmALS3* using the BE4max‐dCas12‐SF01 cytosine base editor system, which was constructed through introducing the E844A mutation in the conserved active site of Cas12‐SF01 (Duan *et al*., 2024) and fusing it to the deaminase Anc689 APOBEC. The cytosine base editor contained a CRISPR RNA (crRNA) transcription box driven by the *GmU6* promoter, and the cassette Anc689 APOBEC‐dCas12‐SF01‐UGI (AncBE4max) driven by the *SlEF1a* promoter (Niu *et al*., 2023). The crRNA1 and crRNA2 targeted *GmALS1* and *GmALS3* respectively (Figure 1a).

**Figure 1:**
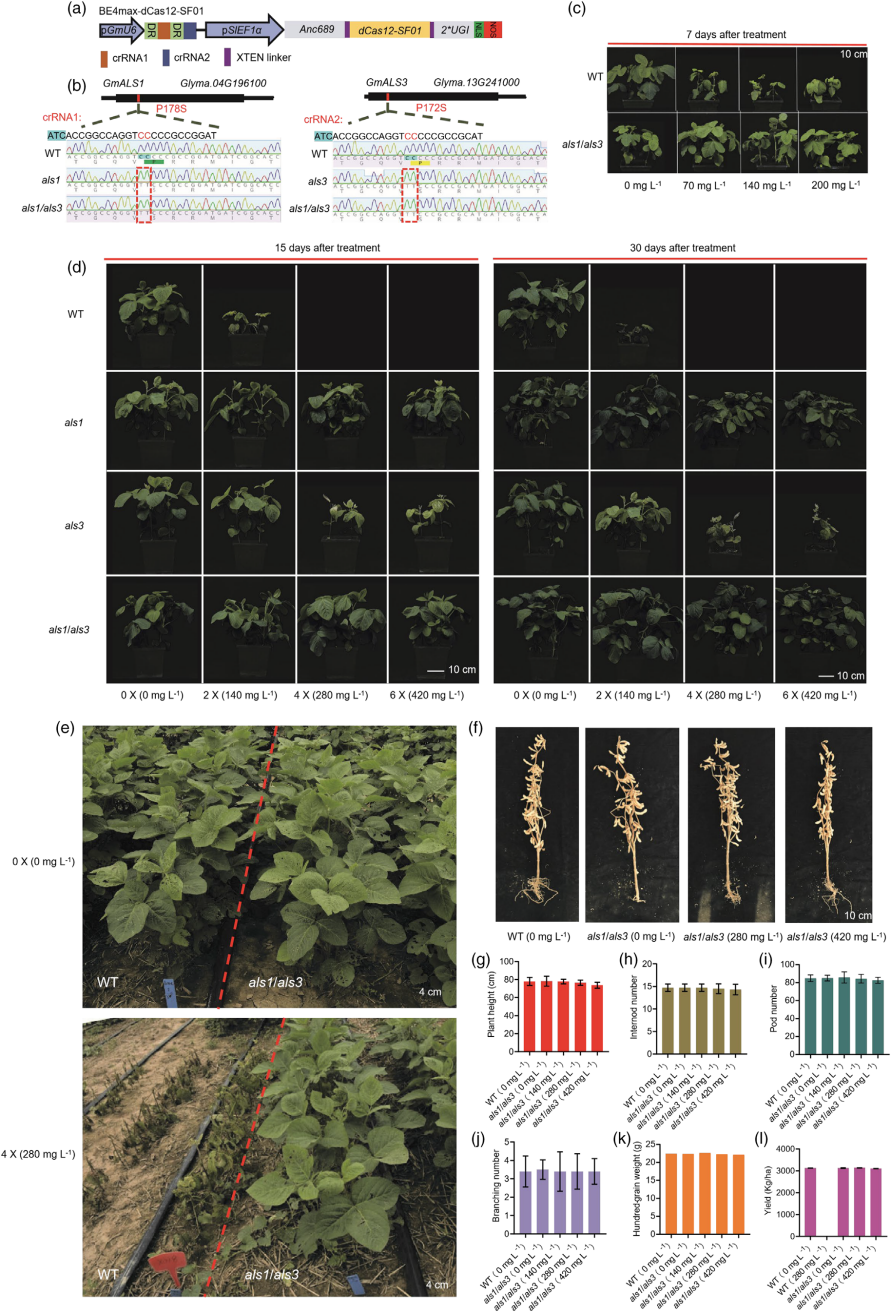
Engineering of herbicide‐resistant soybean plants with the BE4max‐dCas12‐SF01 system. (a) Schematic representation of the binary vector used in this study. NLS, nuclear localization signal; Anc689 APOBEC was directly derived from Wang *et al*. (2019). (b) Genotyping of the als1, als3 and als1/als3 mutants. The ATC (shaded in light blue) at the beginning of the crRNA sites are PAM sites; red boxes indicate C‐to‐T base substitutions. WT, wild type. (c) Morphology of WT and the als1/als3 plants in the greenhouse treated with three concentrations of flucarbazone sodium. (d) Morphology of WT and the als1, als3 and als1/als3 plants 15 or 30 days after treatment with three concentrations of flucarbazone sodium in the greenhouse. (e) Morphology of WT and the als1/als3 plants at the early flowering stage after treatment with 4× flucarbazone sodium in the field. (f) Morphology of WT and the als1/als3 plants treated with three concentrations of flucarbazone sodium at the R8 growth stage. Plant height (g), internode number (h), pod number (i), branching number (j) and hundred‐grain weight (k) of WT and als1/als3 plants. (l) Grain yield of WT and the als1/als3 plants treated with two concentrations of flucarbazone sodium in Jinan (China). Data are presented as means ± SD (*n* = 80 [number of individual plants/genotype] in g–k, 3 [number of areas, each area contains 80 plants/genotype] in l).

We introduced the base editor into the elite soybean cultivar ‘Xudou 18’ through *Agrobacterium tumefaciens*‐mediated transformation. The base‐editing events at the crRNA target sites showed an overall 2.16% editing efficacy (with 9216 explants, we obtained 416 independent T_0_ transformants and 9 of them carried gene‐editing events (4 for *als1*, 3 for *als3* and 2 for *als1/als3*, respectively)). Among the plants harbouring C–T substitutions, we successfully obtained *ALS1* ^*P178S*^, *ALS3* ^*P172S*^ and *ALS1* ^*P178S*^ *ALS3* ^*P172S*^ mutants (referred to as *als1*, *als3* and *als1/als3*, respectively), and potential off‐target sites were examined and no editing events were detected. We obtained transgene‐free stable homozygous *als1*, *als3* and *als1/als3* plants in the T_1_ generation.

We evaluated the herbicide resistance of wild‐type (WT) and *als1/als3* plants grown in a greenhouse by spraying the plants with different concentrations of flucarbazone sodium at the V2 stage. Seven days after the herbicide application, the WT plants were severely damaged while the *als1/als3* plants showed no damage (Figure 1c). We repeated the experiment in the following year and included the *als1* and *als3*. The *als1* and *als3* mutations both conferred some level of herbicide resistance, whereas the *als1/als3* showed no symptoms of herbicide damage (Figure 1d). These results showed that *als1* and *als3* mutations have a synergistic effect that strongly promotes herbicide resistance. We subsequently examined herbicide resistance of the *als1/als3* and WT plants grown in the field. The *als1/als3* plants showed no symptoms of herbicide damage while the WT plants were severely damaged by the herbicide (Figure 1e). The herbicide‐treated *als1/als3* plants showed no differences in plant height, internode number, pod number, branching and hundred‐grain weight from those of the non‐treated WT plants (Figure 1f–k). Furthermore, the productivity of the *als1/als3* plants showed no obvious change compared with that of the WT in field tests (4 m^2^/plot). The yield of the *als1/als3* was not reduced under flucarbazone sodium dosages of 280 and 420 mg L^−1^ compared with 3125 kg/ha for the non‐treated WT (Figure 1l).

Inappropriate dosages (over 2× (120 mg L^−1^) flucarbazone sodium) or uneven application of herbicides by farmers in the field could easily exceed the resistance threshold of crops (Walter *et al*., 2014). Improvement of the herbicide tolerance to meet the requirements of practical breeding applications can overcome these problems. In this study, we successfully established a BE4max editor system that efficiently introduced C‐to‐T conversions in soybean and generated the *als1/als3* mutant, which showed tolerance to 6× (420 mg L^−1^) flucarbazone sodium herbicide (Figure 1d). The identification of the *als1/als3* with 6× flucarbazone sodium herbicide resistance shows the effectiveness of our strategy for breeding herbicide‐tolerant soybeans for real‐world applications.

## Conflict of interest

The authors have declared no conflict of interest.

## Author contributions

Q.N., H.X., J.‐K. Z. and J.Z. contributed to the conceptualization of the project; Q.N., H.X., X.C., M.S., X.W., S.L., K.P. and Y.Z. performed the research; Q.N., H.X. and J.Z. analysed the data; Q.N., H.X., J.‐K. Z. and J.Z. wrote the manuscript. All authors read and approved the final version of the manuscript.

## Data Availability

Data and materials generated from this work will be avaialble upon request. Dr. Jianhua Zhu is fully responsible for the distributions of all materials associated with this article.
